# Investigating Tissue Mechanics *in vitro* Using Untethered Soft Robotic Microdevices

**DOI:** 10.3389/frobt.2021.649765

**Published:** 2021-03-18

**Authors:** Raquel Parreira, Ece Özelçi, Mahmut Selman Sakar

**Affiliations:** School of Engineering, Ecole Polytechnique Fédérale de Lausanne, Lausanne, Switzerland

**Keywords:** soft robotics, mechanobiolgy, plasmonics, hydrogels, microfabrication, 3D tissue constructs

## Abstract

This paper presents the design, fabrication, and operation of a soft robotic compression device that is remotely powered by laser illumination. We combined the rapid and wireless response of hybrid nanomaterials with state-of-the-art microengineering techniques to develop machinery that can apply physiologically relevant mechanical loading. The passive hydrogel structures that constitute the compliant skeleton of the machines were fabricated using single-step *in situ* polymerization process and directly incorporated around the actuators without further assembly steps. Experimentally validated computational models guided the design of the compression mechanism. We incorporated a cantilever beam to the prototype for life-time monitoring of mechanical properties of cell clusters on optical microscopes. The mechanical and biochemical compatibility of the chosen materials with living cells together with the on-site manufacturing process enable seamless interfacing of soft robotic devices with biological specimen.

## 1. Introduction

Recent advances in three-dimensional (3D) culture techniques revolutionized the biomimicry of engineered mammalian tissues. These models enable testing of novel therapeutic agents on human tissues, thus circumventing animal trials, and facilitate the discovery of fundamental biological principles (Yamada and Cukierman, 2007; Deglincerti et al., 2016; Dutta et al., 2017; Low et al., 2020). Microfluidic technology and genetic engineering have already become instrumental in revealing biochemical pathways that play important role in the physiology and disease of engineered tissues (Bhatia and Ingber, 2014; Esch et al., 2015; Matano et al., 2015). To match the precision and versatility of chemical and molecular manipulation techniques, novel mechanical manipulation tools are required. Our understanding of cell mechanics during homeostasis and disease has been garnered from techniques that work on monolayers (Polacheck and Chen, 2016). These studies have shown that cellular state depends on sensing and transduction of mechanical signals (i.e., mechanotransduction), which involve proteins and associated pathways generating intracellular signaling that ultimately give rise to transcriptional programming and changes in cell phenotype (Jaalouk and Lammerding, 2009). The shaping of tissues and maintenance of their physiological activity involve the spatial and temporal regulation of mechanics at multiple scales. Lack of tools hinder the development of the field because applying the engineering methods that are optimized for planar substrates to 3D biological samples poses major challenges.

Mechanical loading of *in vitro* tissues must be performed in a way that is compatible with biochemical manipulation techniques and modern imaging modalities such as confocal and two-photon microscopy. That is to say, robotic micromanipulation tools must operate inside transparent chambers with well-defined chemical composition under physiological pH, temperature, and humidity. Another important specification is maintaining the natural microenvironment of the biological samples during testing as cells are exquisitely sensitive to substrate topography and stiffness, as well as the type and density of ligands presented on the surfaces. The end-effector that is in contact with the samples must be compliant (modulus in the kPa range) with tunable surface chemistry. Finally, considering the inevitable accumulation of proteins and other organics from the serum and cell secretion on the machine parts, the device must be disposable.

Robotic manipulation based on traditional microelectromechanical systems (MEMS) technology deliver fine control over deformation (nm to μm) while providing force measurements with high resolution (nN to μN) (Sun et al., 2003; Beyeler et al., 2007; Engler et al., 2007; Kim et al., 2008). Recently, robotic manipulation tools have been integrated with microfluidic chips for high-throughput automated testing of 3D biological samples (Ito et al., 2016; Sakuma et al., 2019). Although MEMS sensors are very sensitive and actuators are precise, the technology is too expensive to be disposable, the end-effectors do not interface well with biological tissues due to their relatively high stiffness and associated electronics, and the design framework cannot be dynamically adapted to the configuration of the specimen. From materials perspective, hydrogels are ideal for biomanipulation because their high water content and tunable chemistry provide excellent compatibility with living matter (Hoffman, 2012; Li and Mooney, 2016; Özkale et al., 2020). Stimuli-responsive hydrogels provide ample opportunities in the course of building soft microscale actuators and machines for biomedical applications (Beebe et al., 2000; Ionov, 2014; Huang et al., 2016, 2019; Hu et al., 2018; Erol et al., 2019; Koike et al., 2020; Tan et al., 2020). Bulk hydrogel actuators, however, mostly suffer from low stress, poor resolution, and limited strain rate. One way to address these limitations is hierarchically structuring hydrogel nanocomposites using bottom-up manufacturing techniques based on self-assembly. This scalable approach provides means to harness efficient nanoscale energy transduction mechanisms at larger scales (Ding et al., 2016). We have recently introduced cell-sized optomechanical actuators that exhibit mechanical properties (4.8 ± 2.1 kPa stiffness) and performance metrics (relative stroke up to 0.3 and stress up to 10 kPa) that are comparable to that of living muscle cells (Özkale et al., 2019). These actuators use energy from near-infrared (NIR) laser illumination, which is converted into mechanical work by gold nanorods encapsulated inside thermoresponsive nanogels, to provide fine control over actuation with sub-micron spatial resolution at millisecond temporal resolution. We showed that a variety of microscopic artifacts can be constructed with the incorporation of passive hydrogel structures that are directly polymerized around the actuators using digital lithography without additional assembly procedures.

In this work, we describe the design, fabrication, and operation of soft robotic devices for long-term dynamic mechanical loading and characterization of 3D biological samples. The robotic toolkit involves several modules; (1) an actuated flextensional mechanism pushing a piston-like end-effector for spatiotemporally resolved compression, (2) a body frame that is used to position the device inside microfluidic channels or open chambers, and (3) a calibrated cantilever beam for the quantification of tissue stiffness. The fabrication method is based on additive manufacturing where the design of different modules can be adapted according to the specifications of each biological sample on the fly. Upon laser exposure, the soft actuator contracts and the mechanism transforms the contraction into displacement of the piston. The deformation of the cantilever reports the stiffness of the sample. We demonstrate the functionality of the prototype using hydrogel sensor beads with known stiffness and 3D cell aggregates.

## 2. Results

### 2.1. Fabrication and Operation of Machine Components

**Figure 1:**
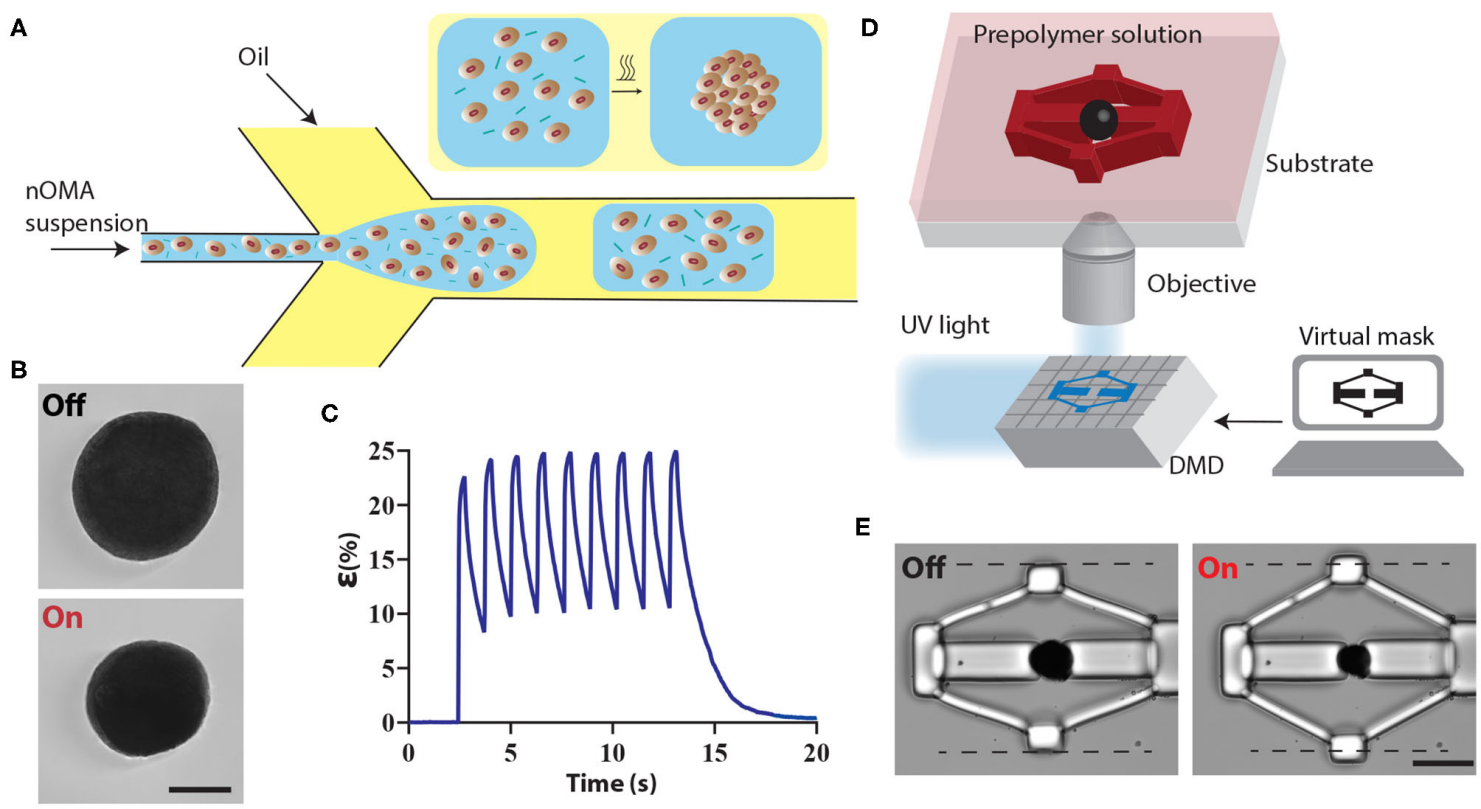
Fabrication and operation of machine components. **(A)** Schematic illustration of the microfluidic colloidal assembly process. An aqueous solution of nOMAs and crosslinker forms the discontinuous phase while the continuous phase comprises the surfactant and oil mixture. The emulsion was collected and heated overnight to facilitate the crosslinking process. **(B)** A representative example showing the fully contracted state of a μOMA upon NIR illumination. The laser power on the sample was adjusted to 10 mW. Scale bar, 100 μm. **(C)** Actuation strain vs. time plot for a 15 mW NIR signal with 260 ms pulse duration at 0.8 Hz. **(D)** The fabrication methodology for building compliant flextensional mechanisms. The projector defines the geometry of the structures polymerizing around the actuators. **(E)** Snapshots from the actuation of a representative mechanism. Scale bar, 50 μm.

The machines were driven by microscale optomechanical actuators (μOMAs) that were fabricated using a template-assisted self-assembly process from nanoscale optomechanical actuators (nOMAs). The nOMAs consist of gold nanorods encapsulated within a thermoresponsive polymer, poly-N-isopropylmethacrylamide (pNIPMAM). We produced monodisperse μOMAs at rates of tens of kHz by means of a microfluidic droplet generator that is based on hydrodynamic flow focusing phenomenon. Figure 1A illustrates the fabrication conditions. The flow rates of the dispersed and continuous phases were controlled by a programmable microfluidic pump. Channel geometry and flow rates together define the size of the droplets, and therefore determine the final size of the actuators.

The μOMAs exhibited drastic deformation upon laser exposure, as shown in Figure 1B. The actuation was highly reversible and could be sustained for at least 10^4^ cycles. Deformation was monitored by measuring the actuation strain, which is defined as percentage change in diameter normalized with respect to the initial diameter (Figure 1C). In principle, the magnitude of actuator collapse can be tuned by changing the illumination profile, i.e., the amplitude, duration, and frequency of the input signal. The maximum strain is determined by the thermoresponsive polymer, which was recorded as 25 ± 2.75 % (*n* = 50) for 200 μm OMAs at 15 mW laser power (measured on the microscope stage). We have previously measured the diffusion coefficient of the actuators as 675 μm^2^·s^−1^ (Özkale et al., 2019), which is two orders of magnitude higher than bulk pNIPMAM hydrogels. The faster diffusion kinetics is a result of the colloidal assembly and the resulting porous architecture of the actuator. Depending on the size of the actuator, the duration of the relaxation phase changes. For example, nOMAs complete the full actuation cycle at the maximum strain within 10 ms. In this work, we used μOMAs that are 100–200 μm in diameter and they completed the full actuation cycle at the maximum strain within 8–15 s.

We fabricated the mechanisms using a maskless projection photolithography method (Chung et al., 2007; Kaynak et al., 2019). The system consists of a programmable digital micromirror device (DMD) module that projects light coming from an ultraviolet LED source through microscope objective according to CAD drawings (Figure 1D). The μOMAs were suspended in a solution of poly (ethylene glycol) diacrylate (PEGDA, 575 kDa) monomer solution supplemented with 20% (v/v) 2-Hydroxy-2-methylpropiophenone (DAROCUR) photoinitiator. UV exposure initiates the free-radical polymerization of structures in the desired form within milliseconds.

We utilized flextensional mechanisms for generating uniaxial compression of biological specimen (Figure 1E). The purpose of the flextensional mechanism is to change the direction of motion (from contraction of the actuator to the extension of the piston) and amplify the displacement generated by the actuator while reducing mechanical advantage as a trade off. The output displacement Δ*y* is a nonlinear function of input displacement Δ*x*.

(1) $$\begin{array}{l} \Delta y=\sqrt{l^{2}sin^{2}\left(\theta \right)-{\left(\Delta x\right)}^{2}+2lcos\left(\theta \right)\Delta x}-lsin\left(\theta \right) \end{array}$$

where *l* is the length of the linkage arms and θ is the nominal linkage angle. We adjusted these two parameters to provide a desired level of compression for the biological sample.

### 2.2. Design and Computational Analysis of the Device

**Figure 2:**
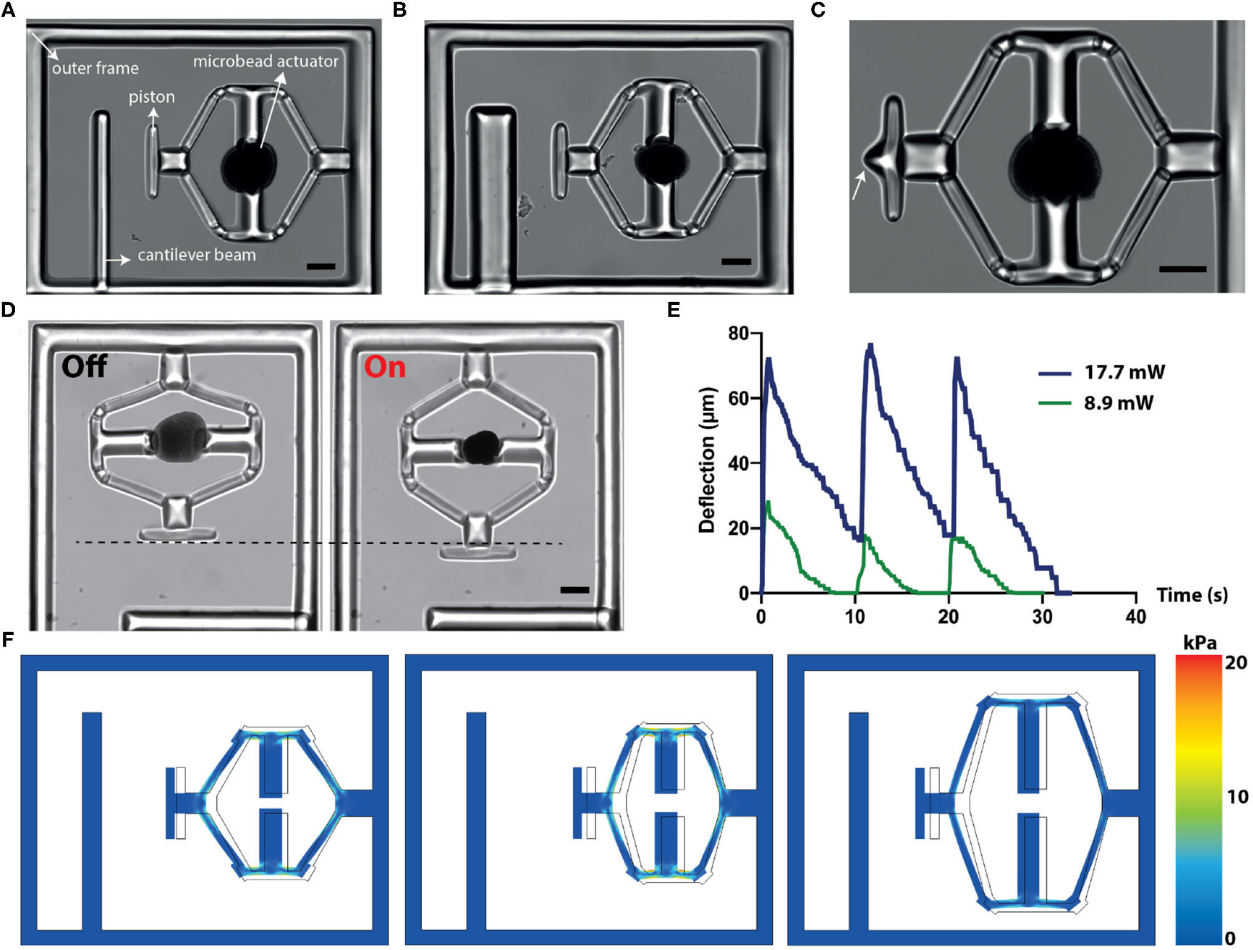
Design, operation, and computational analysis of the compressors. **(A)** A bright-field microscope image of the device showing different modules. **(B)** The cantilever beam geometry can be modified to tune the the sensitivity of the sensor. **(C)** The design of the piston can be modified to tune the applied stress. White arrow points to the sharp tip engineered for localized indentation. **(D)** Temporal sequence of micrographs from a representative actuation cycle. **(E)** The displacement vs. time plot for the piston at different NIR laser power. The signal duration is 500 ms and the actuation frequency is 0.1 Hz. **(F)** Simulation results for different flextensional mechanism designs. The arm angle and length can be modified to tune the piston displacement and stress on the mechanism. Scale bars, 100 μm.

We applied our material system to develop a versatile, remotely controlled biomanipulation platform. Figure 2A shows an image of the compression device, revealing the overall strategy for the simultaneous execution of mechanical loading and characterization operations. There are four major machine components: actuated flextensional mechanism as the driver, end-effector that acts like a piston, cantilever beam for quantifying stiffness, and outer frame. The outer frame is designed to minimize the bending of the actuated structures. The compression device utilized the same flextensional mechanism shown in Figure 1E to convert the contraction of a single μOMA into extension of the arm, which as a result moves the piston forward. The cantilever beam reports the mechanical properties of the compressed specimen. The characteristics of the measurement can be tuned by modulating beam geometry according to the sample properties. Figure 2B shows a prototype with a thicker beam, which enables the device to quantify higher stiffness but with lower sensitivity. The piston, which transmits the force to the sample, can be shaped according to the intended stress profile, as demonstrated by the incorporation of a pointy tip (Figure 2C). Multiple compressors with various different modules were fabricated *en masse* using the programmable features of the DMD module (Supplementary Figure 1).

The displacement of the piston is defined by the actuator size and dynamically controlled by the duration of laser excitation (Figure 2D). In our experiments, the displacement was recorded as high as 170 μm. Figure 2E depicts the piston displacement under the laser illumination with 500 ms pulse duration at 0.1 Hz for two different power levels. We assessed the long-term performance under repeated actuation cycles and confirmed that the displacement did not change after 1,000 cycles.

We built a finite element model of the device to facilitate the design process and report stresses on the beams. Structures were modeled as linear elastic substrates where the empirical value of the Young's modulus (see section 2.4) was used. Experimentally measured actuator displacements were taken as prescribed displacement boundary conditions. Figure 2F consists of simulation results coming from layouts with different arm length and angles, highlighting the relation between geometry and maximum displacement. Moreover, numerical simulations reported the accumulated stress on the actuated beams, which further quantified the device performance.

### 2.3. Heat Transfer During Device Operation

Reliable device performance and long-term biocompatibility of the actuation strategy relies on the following assumption; heating is transient and confined to the actuator. Previous work has shown that the temperature of nOMAs is inversely proportional to the distance from the gold nanorod and heat is dissipated within milliseconds, and thus particles undergo negligible surface heating (Setoura et al., 2013; Liu et al., 2016). Clustering hundreds of nanoheaters together is expected to trap heat inside the μOMA, essentially reducing the laser power required to reach the lower critical solution temperature at which the thermoresponsive polymer goes through a phase transition. On the other hand, negligible surface heating condition may not hold any more.

**Figure 3:**
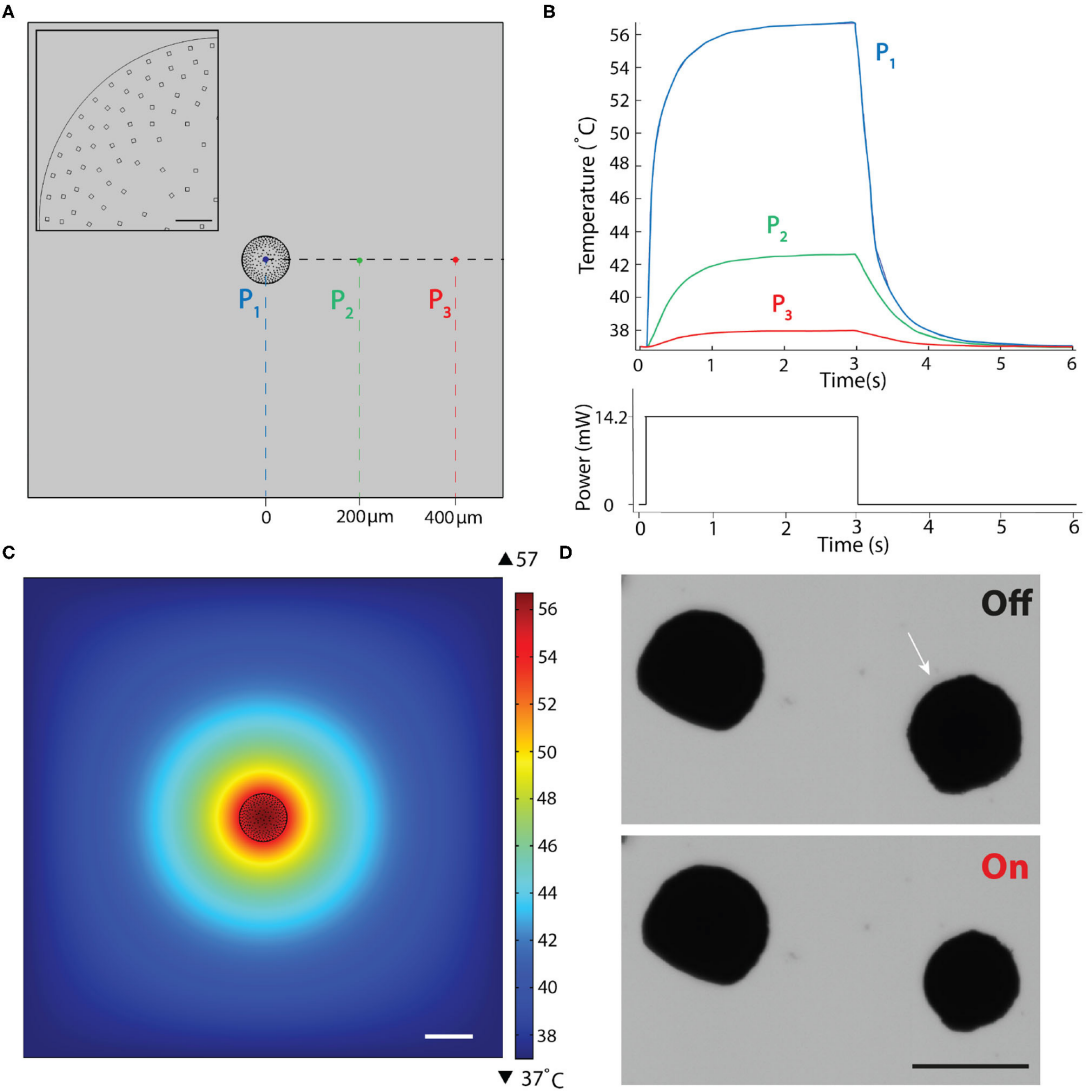
Finite element simulations of the heat transfer during actuation. **(A)** 2D drawing showing the boundary conditions. Inset is a close-up view of the actuator highlighting the nanoheater elements. Scale bar, 10μm. **(B)** Temperature changes over a complete actuation cycle at the three different points shown in **(A)**. The input excitation signal is shown on the bottom plot. **(C)** Temperature as a function of distance from the μOMA irradiated at 14.2 mW laser power at the steady state. Scale bar, 100 μm. **(D)** Selective actuation of a μOMA (denoted with the white arrow) at 14.2 mW laser power in close proximity of another μOMA. Bottom image shows that only the illuminated actuator responds to the signal. Scale bar, 100 μm.

We performed numerical simulations to assess whether heat transfer during the actuation of μOMA could compromise the viability of biological samples. We first calculated the number of nOMAs that would fit into a 400 nm thick cross-sectional cut in the middle of a 100 μm-diameter actuator, assuming close packing of equal particles. Figure 3A shows the geometric configuration of the computational model. Single rectangle shown in the inset of Figure 3A corresponds to a group of 400 nOMAs. Conduction in solids is used to assign the boundary heat source value to each nOMA, which is calculated using the following formula:

(2) $$\begin{array}{l} P=\frac{P_{laser}}{A_{laserbeam}}A_{absorption} \end{array}$$

where *P*_*laser*_ is the measured laser power, *A*_*laserbeam*_ is the area of the laser beam used to actuate the μOMA, *A*_*absorption*_ is the absorption cross-section of one gold nanorod. In the reported simulation results, *P*_*laser*_ = 14.22 mW, *A*_*laserbeam*_ = 1.96·10^−9^ m^2^, and *A*_*absorption*_ = 6,000 nm^2^. μOMA was placed in a 1 mm^2^ water bath, which was taken to be large so that we could eliminate potential issues associated with diffusion. Note that we assigned a fixed boundary temperature of 37°C to the walls of the bath. The software solved the following equation:

(3) $$\begin{array}{l} d_{z}\rho C_{p}\frac{\partial T}{\partial t}+d_{z}\rho C_{p}\text{u}\cdot \nabla T+\nabla \cdot \text{q}=d_{z}Q+q_{0}+d_{z}Q_{ted} \end{array}$$

where *Q*_*ted*_ is the thermoelastic damping coefficient and *d*_*z*_ is the thickness of domain in the out-of-plane direction. We approximated the shape as a disk with thickness of 400 nm, which is the diameter of a single nOMA. *Q*_*ted*_ is the thermoelastic damping which represents the thermoelastic effects in solids. The conductive heat flux, **q**, is given by **q** = −*d*_*z*_*k*∇*T*.

In our material system, plasmon heating phenomenon is expected to stabilize at a certain temperature for a given laser power. To experimentally demonstrate this property, we continuously actuated beads for an hour at a given laser power and verified that the level of contraction stayed the same (Supplementary Figure 2). Considering the transient response, the settling time for the temperature to reach its equilibrium value depends on the laser power as well as the boundary conditions. Taking this dynamics into account, in our simulations, we applied a laser pulse as an input to the simulation that was long enough for the system to reach steady state. Figure 3B shows the evolution of temperature at three different points that were denoted in Figure 3A. Figure 3C shows the temperature distribution around the actuator at the equilibrium. The highest temperature was reported as 56.7°C at the core of the actuator for the given laser power (*P*_*laser*_ = 14.2 mW), which dissipated with a relatively steep curve. The steady-state temperature at locations 200 and 400 μm away from the surface of the μOMA were 42.6 and 37.9°C, respectively.

We experimentally validated the implications of this analysis by placing the actuators close to each other at various distances. Figure 3D shows the selective actuation of a μOMA in the vicinity of another μOMA. The actuator that was placed 150 μm away from the activated one did not show any response. We only observed reaction from the non-activated actuator once the distance between the actuators were <50 μm. These observations showed that numerical analysis was overestimating the temperature. Taken together, we have a framework to design the soft robotic device and tune the control parameters so that the temperature around the biological specimen never exceeds the tolerable levels. In the experiments presented in section 2.5, the spheroids were placed 500 μm away from the actuator to protect the samples from overheating.

### 2.4. Calibration of the Sensing Probe

**Figure 4:**
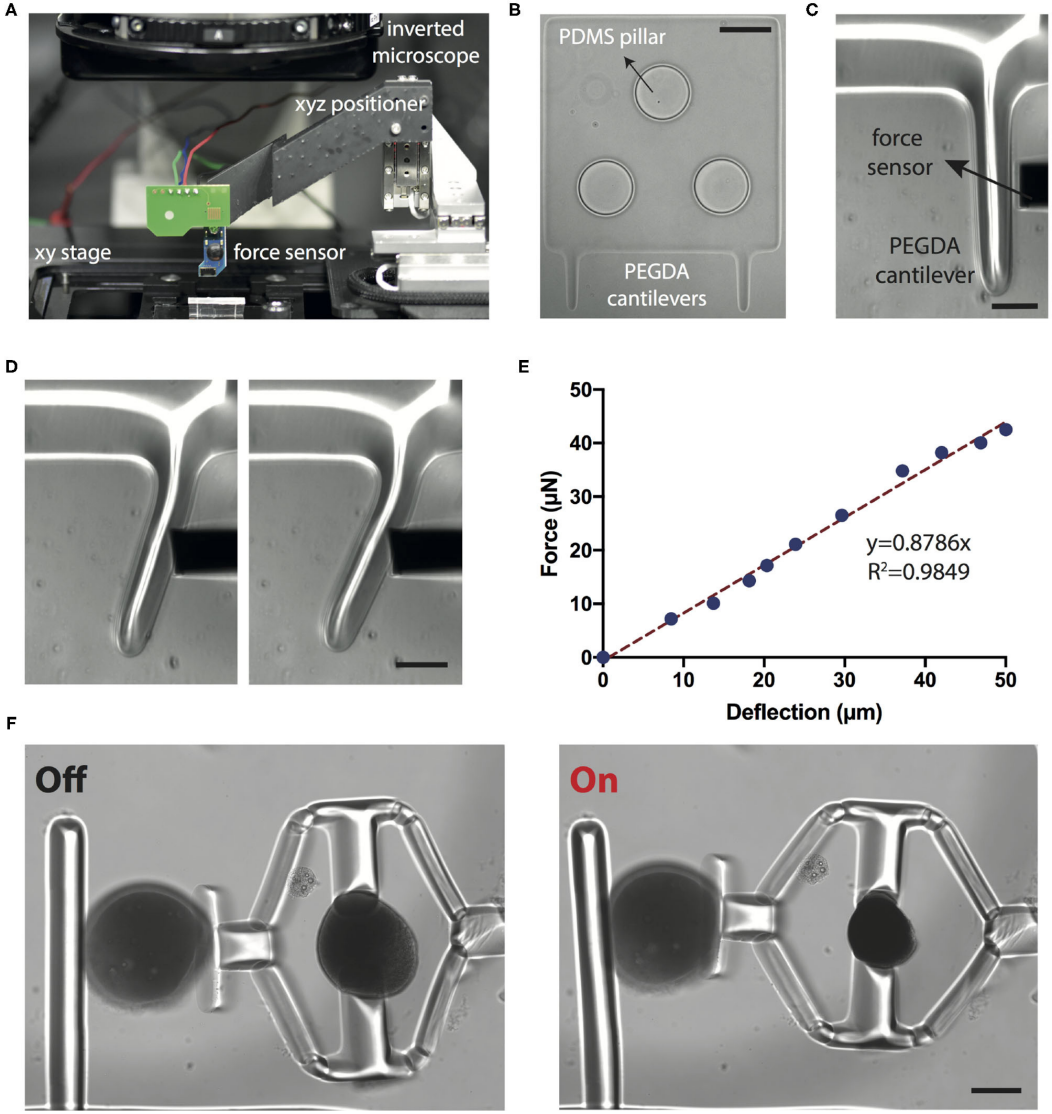
Calibration of the sensing probe. **(A)** The position of the MEMS force sensor is controlled by a motorized 3-axis micromanipulator while the sample is translated on a plane using the microscope stage. **(B)** The base of the cantilever beams is polymerized directly around the PDMS micropillars using the digital maskless lithography system. The pillars stabilize the base and keep it stationary during mechanical loading. Scale bar, 100 μm. **(C)** Close-up of the cantilever beam and the tip of the sensor. Scale bar, 50 μm. **(D)** Snapshots from a representative indentation experiment. Scale bar, 50 μm. **(E)** A representative force vs. deflection curve. The slope of the linear fit is used for calculating the bending modulus. **(F)** Representative images from a compression test for PAAm beads. Scale bar, 100 μm.

The stiffness measurements rely on the calibration of the cantilever beam, which was performed using a commercially available capacitive MEMS force sensor. The force sensor was mounted on a three-axis motorized micromanipulator while the workspace was monitored using an optical microscope (Figure 4A). The base of the PEGDA cantilevers were polymerized around poly-(dimethylsiloxane) (PDMS) pillars that were microfabricated on the surface of a glass chamber to avoid motion during loading (Figure 4B). The sensor is capable of measuring forces perpendicular to the sensor's axis, which allows for the monitoring of both the sensing-probe tip and the sample under the microscope (Figure 4C). The beam was progressively deformed with a speed of 1 μm·s^−1^ (Figure 4D). Force vs. deflection curve shown in Figure 4E was obtained using the calibration settings (i.e., coefficient for voltage to force conversion) provided by the manufacturer. We calculated the Young's modulus (*E*) of PEGDA structures as 800 ± 85 kPa (six different beams and six independent measurements from each beam) using the Euler-Bernoulli beam equation:

(4) $$\begin{array}{l} E=\frac{F\left(3l-x\right)x^{2}}{6I\delta_{max}} \end{array}$$

where *F* is the force, *l* is the length and *I* is the second moment of inertia of the beam, *x* is the distance from the base of the beam to the location of loading, and δ_*max*_ is the maximum deformation of the beam.

We next tested the accuracy of our measurements using polyacrylamide (PAAm) microgel beads. Previous work has shown that PAAm microgel beads can be used as standardized calibration samples for mechanical measurements (Dolega et al., 2017; Girardo et al., 2018). Beads with controlled size and elasticity can be fabricated by tuning the pre-gel composition (Girardo et al., 2018). Importantly, using this protocol, microgels with elasticity in the range of tissue relevant mechanical properties can be fabricated. We fabricated microgels with elasticity ranging from 1.92 ± 0.46 kPa (*n* = 10) to 9.19 ± 3.82 kPa (*n* = 10) by varying the monomer (PAAm) and crosslinker (BIS) concentrations. Young's modulus was measured using the same force sensor and these values are in accordance with AFM characterization data (Girardo et al., 2018).

PAAm beads were placed into the compression device using a micromanipulator and the laser power was increased with 0.8 mW increments. The deformation of the cantilever probe along with the displacement of the piston were measured from the microscope images, which together quantify the deformation of the PAAm bead (Figure 4F). Force acting on the cantilever beam were calculated using Euler-Bernoulli beam theory and experimentally measured Young's modulus. The calculated force is assumed to be the same as the one applied on the PAAm bead. The Young's modulus of the beads were then calculated using Hertzian half space contact mechanics model:

(5) $$\begin{array}{l} \;\delta =\frac{3\left(1-\nu^{2}\right)F}{4Ea}-\frac{f\left(a\right)F}{\pi E},\\ f\left(a\right)=\frac{2\left(1+\nu \right)R^{2}}{{\left(a^{2}+4R^{2}\right)}^{3/2}}+\frac{1-\nu^{2}}{{\left(a^{2}+4R^{2}\right)}^{1/2}}. \end{array}$$

where *R* is the radius of the sphere, ν is Poisson's ratio (taken as 0.49), δ is the compressive displacement, *f* is the force, and *a* is the radius of contact area. Young's modulus can be extracted from the relationship between δ and *f* for values satisfying the condition δ/*R* ≤ 0.2. This model accommodates for large deformations (Kim et al., 2010).

In this work, we only made measurement for one material composition. The Young's modulus of the PAAm beads with 40% (w/w) PAAm, 2% (w/w) BIS was measured as 5.75 ± 1.63 kPa (*n* = 6). At this composition, the Young's modulus was measured as 7 ± 2.15 kPa (*n* = 8) by the force sensor shown in Figure 4C. Preliminary results demonstrate the feasibility of making quantitative measurements using an all-hydrogel, remotely actuated compliant mechanism. However, further characterization with many samples is required to provide a comprehensive calibration data. The material of choice is not limited to soft hydrogels that swells and adsorbs proteins. We fabricated devices from another polymer, Trimethylolpropane ethoxylate triacrylate (TPETA, 692 kDa), that is elastic and protein repellent (Supplementary Figure 3). Depending on the application, different modules can be fabricated from different photopolymerizable polymers.

### 2.5. Biomechanical Characterization

**Figure 5:**
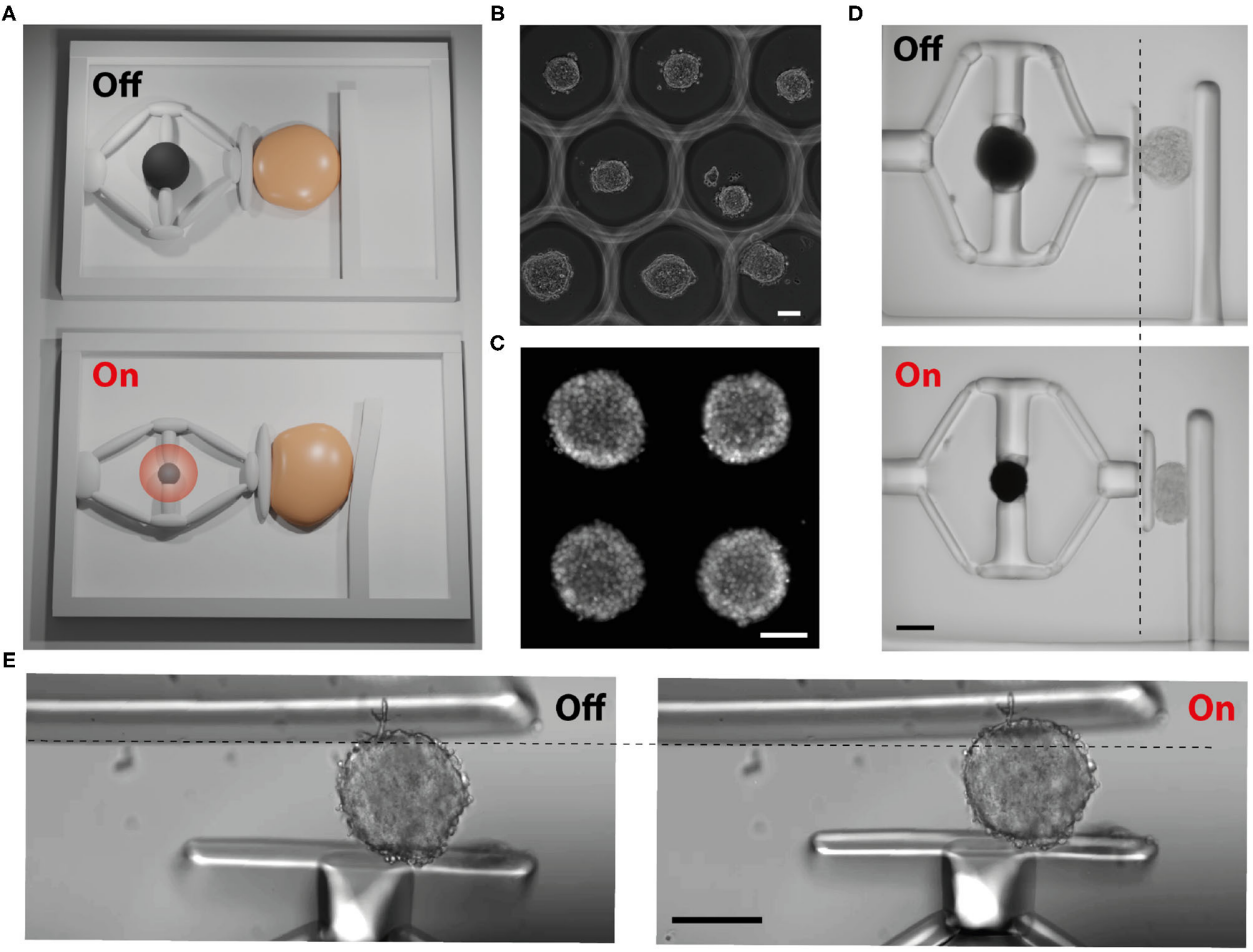
Biomechanical characterization of 3D tissue samples. **(A)** Schematic depiction of the working principle (not to the scale). Upon application of NIR illumination, the monolithic compliant mechanism deforms and the piston compresses the sample. The cantilever beam reports the sample stiffness. **(B)** Phase-contrast image shows high-throughput fabrication of spheroids using hydrogel wells. **(C)** Fluorescent image of an array of self-assembled spheroids labeled with a DNA dye. **(D)** Snapshots from a representative compression experiment where the deflection of the beam is not obvious. **(E)** A compression device with a more sensitive cantilever beam. The beam deflects during the loading of the sample, reporting its stiffness. Scale bars, 100μm.

As a proof-of-concept demonstration, we tested the feasibility of compressing cell clusters (spheroids). The overall simultaneous mechanical loading and characterization concept is illustrated in Figure 5A. We have previously verified the biocompatibility of the materials by performing cell viability assays (Özkale et al., 2019). Spheroids were self-assembled from human embryonic kidney (HEK) cells using a high-throughput microengineered cell culture device (Figure 5B). Cells adhere to each other and form a compact 3D tissue with a spherical shape over two days of culture (Figure 5C). They were then transferred to the chamber where the devices were operated.

Figure 5D shows a representative example for the compression of the spheroids. A single spheroid was placed between the piston and the cantilever beam using a micromanipulator. We recorded a compressive strain up to 40% with a device that had a cantilever with 70 μm thickness and an μOMA with 160 μm diameter. We observed a slight deflection on the cantilever, corresponding to a few μm displacement. The beam was too stiff for making a reliable measurement but this experiment showed how much a spheroid could be compressed using the current prototype as the force was primarily harnessed to load the sample. We fabricated a compressor device with a thinner cantilever, having a thickness of 20 μm. This time the deflection of the beam was obvious (Figure 5E). The Young's modulus of the sample was measured as 2.45 ± 0.46 kPa (*n* = 6). The geometry and stiffness of the cantilever beam must be tuned carefully according to the characteristics of the sample, which may require prior measurements done with an electronic probe or high-throughput screening using various cantilever designs.

### 2.6. Integration With Microfluidic Systems

Microfluidic cell culture devices push the biomimicry of engineered tissues to the next level by providing long-term perfusion, introduction of chemicals and cells in a dynamic fashion, and notably, improving the reproducibility and throughput via automation (Nikolaev et al., 2020; Novak et al., 2020; Schuster et al., 2020). We developed an integrated microfluidic solution to showcase that our fabrication and actuation paradigm is compatible with these devices. We fabricated an open PDMS chamber with docking stations on its walls for assembling the compressors (Supplementary Figure 4). The actuation modules were transferred to this chamber in the same hydrogel prepolymer solution, positioned at target docking stations using a micromanipulator, and anchored to the walls with an additional projection photolithography step. This final step ensured that the compressors did not rotate or detach during sample loading and actuation. The sensing module was fabricated on the other side of the chamber during the manufacturing of the base. The chamber was closed once the biological samples were placed in position. As an alternative to the docking site approach, the base could be polymerized around pillars as shown in Figure 4B, which would reduce the area of the blueprint.

## 3. Discussion

Maskless optofluidic photopolymerization process enables the manufacturing of custom shaped functional microparts that are physically connected to the actuation units. With the presented methodology, we can *in situ* fabricate arrays of devices inside microfluidic chips. By taking advantage of laminar flow and pneumatic valves, the microfluidic chip can be programmed to place biological samples at designated compartments. This way, we can build a fully automated, high-throughput mechanochemical testing platform. The blueprint for the chassis and machine components are highly re-configurable, which allows rapid design and prototyping of a variety of different soft robotic systems.

Forces applied by the device are within physiological levels (on the order of μN). The deformation and stress profiles can be further modulated by changing the design of the flextensional mechanism and the shape of the piston. This methodology allows real-time monitoring of changes in gene expression and protein signaling in response to controlled mechanical loading. Cantilever beam simultaneously provides real-time measurements of elasticity. Together, we can monitor and perturb both physical and biochemical properties of the tissues. While the cantilever based measurements are not as sensitive as electrostatic comb drives, the sensitivity is acceptable for capturing major changes in tissue mechanics. We will further tune the geometry and material properties of the system for optimizing the measurement conditions.

Projection lithography is convenient and versatile yet the design of the mechanisms are limited to extruded shapes. The polymers that we used, on the other hand, have already been adapted to 3D nanoprinting using two-photon polymerization (Hippler et al., 2019, 2020; Kaynak et al., 2020). Printing mechanisms around the actuators is one interesting avenue. An alternative solution is to push the printing concept to the next level. We have recently shown that the nOMAs could be assembled on-demand by harnessing thermocapillary flows (Parreira et al., 2020). Thus, it is conceivable that the whole machine can be manufactured *in situ* using direct laser writing. With the resolution provided by printing, machines can perform dexterous manipulation of biological samples.

## 4. Materials and Methods

All reagents were purchased from Sigma Aldrich and used as received, unless stated otherwise.

### 4.1. Synthesis of Actuators

The gold nanorods were synthesized following a previously published protocol (Liu et al., 2016). Transmission electron microscopy, UV-Vis spectroscopy and dynamic light scattering were used to characterize the morphology, structure and optical response of the nanoactuators. The microfluidic devices were fabricated from poly-(dimethylsiloxane) (PDMS) using soft lithography. The discontinuous phase was composed of 16 mg·ml^−1^ nanoactuator suspension and 20% (v/v) glutaraldehyde (GA), while the continuous phase was a solution of 2% (v/v) surfactant (Picosurf 5%) in fluorinated oil (Novec 7500). The emulsion was heated overnight in silica-treated glass vials at 65°C to promote the nanoparticles crosslinking through an amine-aldehyde condensation reaction. The resulting samples were purified with 20% 1H,1H,2H,2H-Perfluoro-1-octanol (PFO, 97%) in fluorinated oil and washed three times with ethanol and three times with Milli-Q water to remove unreacted GA.

### 4.2. Laser Actuation

All experiments were performed using a motorized inverted microscope (Nikon Ti-E) and images were captured with an ORCA-Flash4.0 CMOS camera (Hamamatsu). A 785 nm laser (110 mW, Thorlabs) beam coupled to the microscope provided NIR illumination. The laser illumination was modulated using a custom LabView program. During biological manipulation, the devices were submerged in culture medium supplemented with HEPES solution and the chamber was kept at physiological conditions using an environmental chamber (Life Imaging Services). The videos were captured at full pixel resolution (2,048 × 2,048) with frame rates ranging from 33 to 200 fps depending on the requirement of the experiment. A program based on an edge-detection algorithm in Matlab (Mathworks, MA) was used to measure the deformation from time-lapse videos.

### 4.3. Digital Maskless Lithography

Maskless projection photolithography is performed with a programmable digital micromirror device module (Andor Technology) connected to the microscope. The digital projector contains an 800 × 600 micromirror array and operated with a minimum exposure time of 50 μs at a maximum frame rate of 5,000 fps. The platform projects light provided by an ultraviolet LED source (365 nm) using a computer-aided design (CAD)-based digital blueprint, which initiates subsequent free-radical polymerization of photosensitive materials. 2D drawings defined the planar shape of the structures, whereas the height was simply controlled by the channel size. Overlapping regions between the mechanism and the actuators were created in the polymerization process to ensure a mechanically stable connection and efficient transmission of forces. The modulated beam was projected through a 10X microscope objective onto the substrate where we had actuators suspended in the hydrogel prepolymer solution.

### 4.4. MEMS Force Sensor Measurements

A commercial MEMS force sensor (FT-S1000-LAT, FemtoTools) with a resolution of 0.05 μN was mounted on a motorized XYZ micromanipulator (SLC-2040, SmarAct GmbH) using a 3D printed adapter. The force sensor was positioned manually using the corresponding controller while being visually monitored using the microscope. The force sensor was powered by a programmable linear power supply (Keysight E3631A), and output was measured using a precision multimeter (Keysight 34465A).

### 4.5. Synthesis of Polyacrylamide Beads

The polyacrylamide (PAAm) beads were synthesized following a protocol from the literature (Girardo et al., 2018) with some modifications. The reaction solution was obtained by mixing 0.3% (w/v) ammonium persulfate (APS) with different amounts of acrylamide (AAm) and bis-acrylamide (BIS) in 10 mM Tris buffer. This solution was added to the continuous phase composed of mineral oil with 5% (v/v) surfactant Tween 80. The emulsion was stirred at 800 rpm and purged with N_2_ for a minimum of 15 min. Subsequently, 300 μL of the catalyzer Tetramethylethylenediamine (TEMED) was added drop-wise. The obtained beads were cleaned through centrifugation with ethanol and Milli-Q water.

### 4.6. Formation of Spheroids

HEK293T cells were cultured in Dulbecco's modified Eagle medium (DMEM/F-12) supplemented with 10% fetal bovine serum and 1% penicillin/streptomycin. Cells were passaged upon achieving confluency at a 1:4 ratio using 0.25% Trypsin-EDTA and discarded after 20 passages. All experiments were done using cells tested for mycoplasma negative. A high-throughput device with microengineered hydrogel films on the bottom of conventional multiwell plates was used to initiate spheroid formation (Brandenberg et al., 2020). The size of spheroids was controlled with the initial cell number. In this work, to produce spheroids with 150 μm in average, 1,000 cells were used. The spheroids were transferred to the device after 32 h of culture. The nuclei were labeled using Hoechst 33,342 stain.

### 4.7. Simulations

Structural analysis and calculations of the temperature distribution were performed by finite element simulation (Comsol Multiphysics, Burlington MA). The heat capacity and thermal conductivity of the polymer hydrogel is assumed to be equivalent to that of water (Liu et al., 2016). Smallest mesh element size is used as 0.125 μm and biggest element size is chosen as 37 μm with maximum element growth rate of 1.25. Accuracy of the mesh was ascertained through a mesh refinement study.

## Data Availability Statement

The original contributions presented in the study are included in the article/Supplementary Material, further inquiries can be directed to the corresponding author/s.

## Author Contributions

MSS designed the study. RP performed the experiments. RP and EÖ analyzed the data. EÖ performed the finite element simulations. RP, EÖ, and MSS wrote the manuscript. All authors have given approval to the final version of the manuscript.

## Conflict of Interest

The authors declare that the research was conducted in the absence of any commercial or financial relationships that could be construed as a potential conflict of interest.
