# Supplementary material for: Investigating Tissue Mechanics in vitro Using Untethered Soft Robotic Microdevices
Source: Front Robot AI. 2021 Mar 18;8:649765. doi: 10.3389/frobt.2021.649765 (PMC8044975; doi:10.3389/frobt.2021.649765)
Supplement: Supplementary file 1 [file Data_Sheet_1.pdf]

Supplementary Material

SUPPLEMENTARY TABLES AND FIGURES

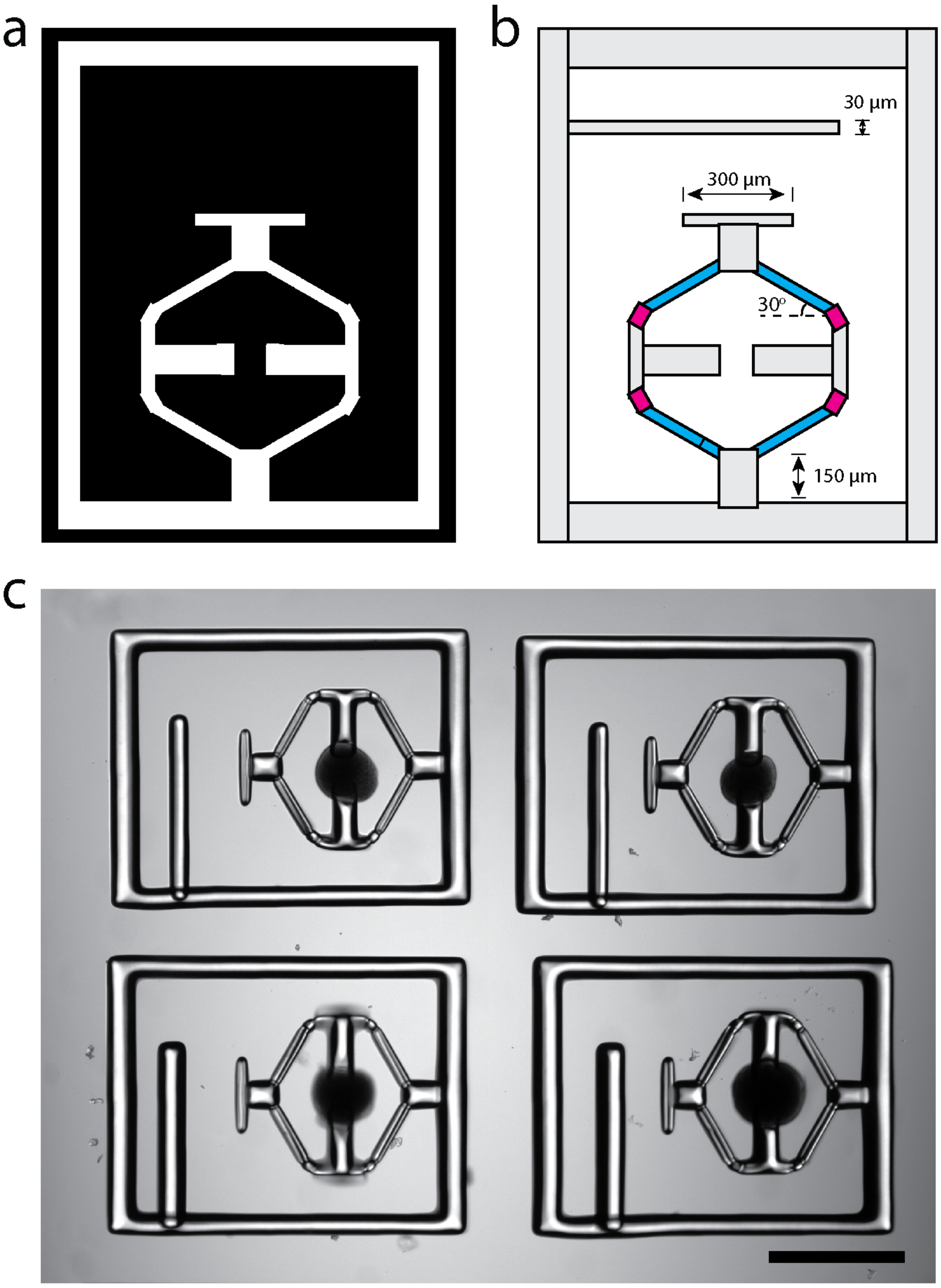

Figure S1. Rapid prototyping of compression devices using projection lithography. (a) A representative CAD drawing of the device. Single exposure using this digital mask polymerizes the mechanism as a whole. (b) Alternatively, the mechanism is divided into different regions (shown with different colors) and flexible beams are fabricated with separate illumination to avoid over-polymerization. (c) Devices are fabricated en masse. Sensing probes with two different sensitivities are shown on the top and bottom rows. Scale bar, 500 μm.

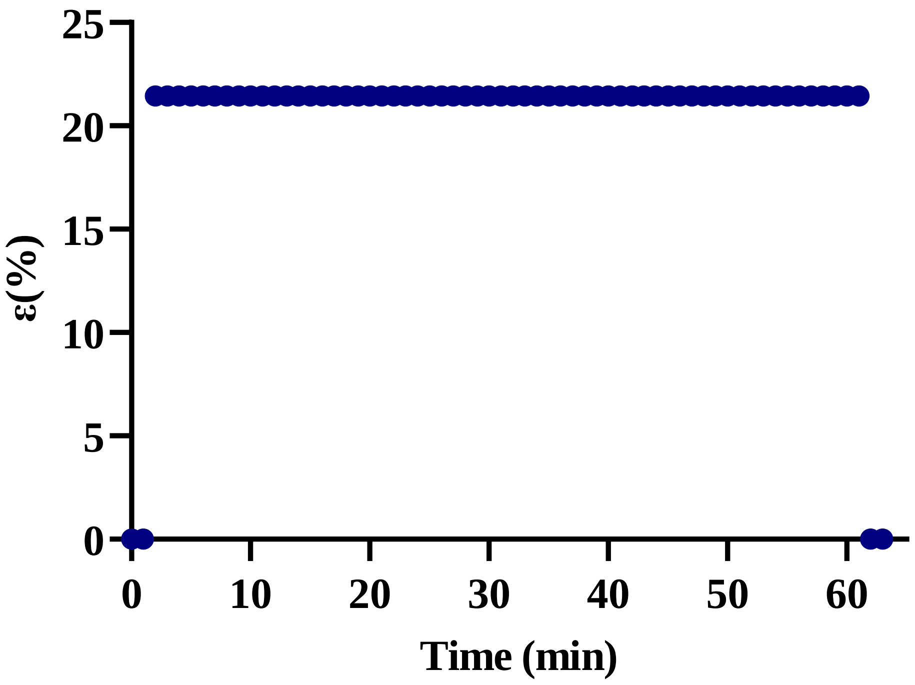

Figure S2. The deformation of the microactuator does not change over time for a given laser power. The strain generated by a microactuator under continuous 15 mW laser illumination is measured every minute over an hour.

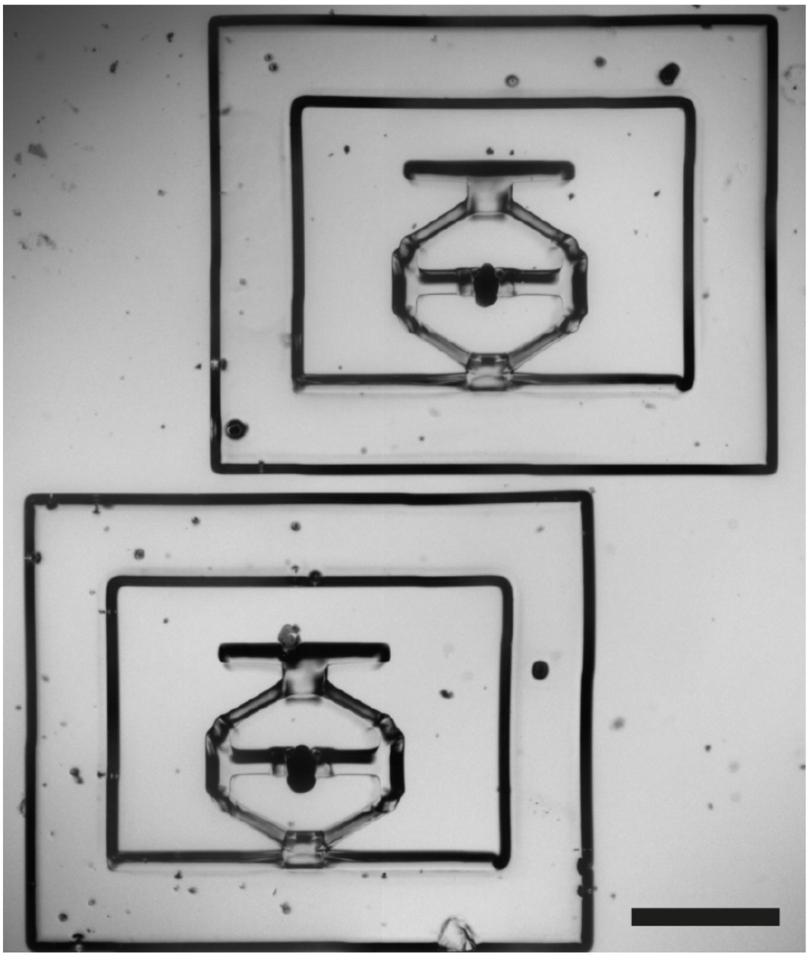

Figure S3. Flexible devices fabricated from TPETA polymer. Scale bar, 500 μm.

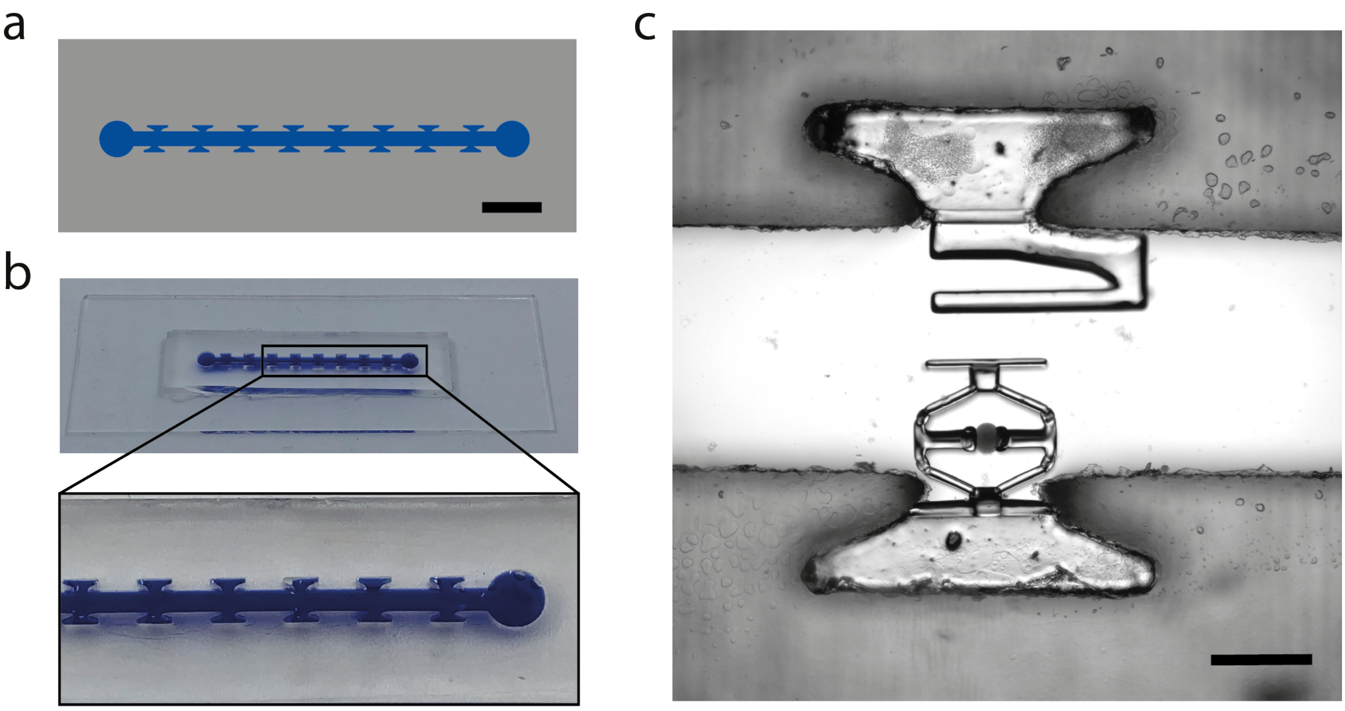

Figure S4. Assembly of devices inside microfluidic systems. (a) CAD design of the microfluidic device. Scale bar, 5 mm. (b) Photographs of the microfluidic chip. The engraved docking stations are highlighted with blue ink. (c) Representative example of a soft robotic device assembled inside the channel. The compression module was fabricated elsewhere and transported to the channel while the sensing module and the base were polymerized inside the channel. Scale bar, 500 μm.
